# Delirium in COVID-19: epidemiology and clinical correlations in a large group of patients admitted to an academic hospital

**DOI:** 10.1007/s40520-020-01699-6

**Published:** 2020-09-18

**Authors:** Andrea Ticinesi, Nicoletta Cerundolo, Alberto Parise, Antonio Nouvenne, Beatrice Prati, Angela Guerra, Fulvio Lauretani, Marcello Maggio, Tiziana Meschi

**Affiliations:** 1 Geriatric-Rehabilitation Department, Azienda Ospedaliero-Universitaria di Parma, Via Antonio Gramsci 14, 43126 Parma, Italy; 2 Department of Medicine and Surgery, University of Parma, Parma, Italy

**Keywords:** Coronavirus pneumonia, SARS-CoV-2, Delirium, Multimorbidity, Dementia

## Abstract

**Background:**

Delirium incidence and clinical correlates in coronavirus disease-19 (COVID-19) pneumonia are still poorly investigated.

**Aim:**

To describe the epidemiology of delirium in patients hospitalized for suspect COVID-19 pneumonia during the pandemic peak in an academic hospital of Northern Italy, identify its clinical correlations and evaluate the association with mortality.

**Methods:**

The clinical records of 852 patients admitted for suspect COVID-19 pneumonia, defined as respiratory symptoms or fever or certain history of contact with COVID-19 patients, plus chest CT imaging compatible with alveolar-interstitial pneumonia, were retrospectively analyzed. Delirium was defined after careful revision of daily clinical reports in accordance with the Confusion Assessment Method criteria. Data on age, clinical presentation, comorbidities, drugs, baseline lab tests and outcome were collected. The factors associated with delirium, and the association of delirium with mortality, were evaluated through binary logistic regression models.

**Results:**

Ninety-four patients (11%) developed delirium during stay. They were older (median age 82, interquartile range, IQR 78–89, vs 75, IQR 63–84, *p* < 0.001), had more neuropsychiatric comorbidities and worse respiratory exchanges at baseline. At multivariate models, delirium was independently and positively associated with age [odds ratio (OR) 1.093, 95% confidence interval (CI) 1.046–1.143, *p* < 0.001], use of antipsychotic drugs (OR 4.529, 95% CI 1.204–17.027, *p* = 0.025), serum urea and lactate-dehydrogenase at admission. Despite a higher mortality in patients with delirium (57% vs 30%), this association was not independent of age and respiratory parameters.

**Conclusions:**

Delirium represents a common complication of COVID-19 and a marker of severe disease course, especially in older patients with neuropsychiatric comorbidity.

## Introduction

Neurologic manifestations are increasingly described as part of the clinical picture of patients with moderate or severe coronavirus disease-19 (COVID-19), either at the onset or during its course [1, 2]. Among these, delirium, defined as a disturbance of consciousness or cognitive function with acute onset and fluctuating course, is widely known as one of the commonest complications of hospitalization in older patients also outside the context of the COVID-19 pandemic [3].

Infections are frequently associated with delirium in older patients, especially if they imply alterations of physiological parameters and laboratory tests and are treated with the use of invasive devices [4–6]. These precipitating factors are common in patients hospitalized with pneumonia [7] and have also been observed in patients with moderate or severe COVID-19 [8]. In this disease, massive activation of inflammation with cytokine storm, endothelial damage, increased oxidative stress, hypoxemia and, probably, direct infection of the Central Nervous System (CNS) by Severe Acute Respiratory Syndrome Coronavirus-2 (SARS-CoV-2) can promote the onset of encephalopathy, with delirious manifestations especially in older frail multimorbid subjects and in those with a history of dementia [8–10].

The older population has been particularly vulnerable to SARS-CoV-2 infection, frequently developing severe forms of COVID-19 with high mortality [11, 12]. In spite of this epidemiology, at the moment of writing the association of COVID-19 with delirium has been investigated only sporadically [13], with some anecdotal reports of patients presenting delirium as the first, and sometimes only, sign of COVID-19 [8, 14, 15]. Thus, the incidence and clinical correlations of delirium during hospital stay for COVID-19 are still poorly known. Moreover, the impact of delirium on mortality, which is well established in ordinary practice [16], is still unknown in the context of COVID-19.

The objective of this retrospective study was to assess the incidence of delirium in a large number of patients hospitalized for COVID-19 in Northern Italy, verify its clinical correlations and determine its impact on in-hospital mortality.

## Methods

### Study population and setting

This study was conducted at Parma University-Hospital, a 1200-bed teaching hospital with a primary catchment area of 305,582 inhabitants in Northern Italy. Since February 28, 2020, the emergence of COVID-19 in Northern Italy has forced our institution to face a massive overflow of patients seeking care for suspect COVID-19. Diagnostic routes dedicated to suspect COVID-19 patients, from the Emergency Department (ED) to general and Intensive Care Unit (ICU) wards, were rapidly instituted [17]. These routes included an initial clinical evaluation in a respiratory pre-triage area of the ED, chest Computed Tomography (CT) scan, and admission to COVID-19-dedicated wards for all patients with CT signs compatible with the presence of alveolar-interstitial pneumonia [17]. SARS-CoV-2 testing was performed immediately after admission to wards [17].

The Geriatric-Rehabilitation Department of our University-Hospital was entirely reconverted to COVID-19 unit, admitting a large number of patients who had just undergone chest CT testing and who showed clinical and radiological features compatible with moderate or severe SARS-CoV-2 infection. The wards of the Department were also equipped with non-invasive ventilators and subintensive care devices, to reduce the need for ICU beds. During the pandemic peak, the medical staff of the Department included also non-geriatric and non-internist specialists, but the procedures were continuously supervised by the ward usual staff and remained consistent with those adopted before the emergency.

We retrospectively analyzed the clinical records of 852 patients admitted to this clinical setting in March and April, 2020. Inclusion criteria were age ≥ 18, admission from the ED for suspect COVID-19, and absence of terminally-ill state at the moment of admission. All patients still hospitalized at the moment of data collection were excluded from the study.

Suspect COVID-19 was defined as presence of respiratory symptoms and/or fever and/or certain history of contact with SARS-CoV-2 positive patients, plus chest CT imaging showing abnormalities compatible with the presence of alveolar-interstitial pneumonia.

### Data collection

From each clinical record, we retrieved data on patient age, gender, symptoms and their duration prior to ED assessment, functional performance, comorbidities, number of chronic medications, baseline vital signs, laboratory tests and chest imaging. Vital signs included blood pressure, respiratory rate, heart rate, oxygen saturation, oxygen flow support and temperature, and were measured at the moment of ward admission. Laboratory tests were performed within six hours from ward admission and included blood cell count, creatinine, urea, sodium, potassium, C-reactive protein (CRP), procalcitonin, aspartate and alanine-aminotransferase, lactate-dehydrogenase, creatine-phosphokinase, D-dimer, fibrinogen, activated partial thromboplastin time (APTT) and prothrombin time (PT). The results of baseline arterial blood gas analysis were also collected. The extension of chest CT abnormalities in lung parenchyma was also recorded, in compliance with the radiological protocol adopted at our institution for the COVID-19 emergency [18].

The presence of delirium during stay in our ward was carefully checked after review of daily reports and charts on clinical records. The presence of delirium was classified on a clinical basis, in compliance with the criteria of the Confusion Assessment Method (CAM) tool, shortened version: acute onset, inattention, impaired consciousness, disorganized thinking and fluctuating course [19]. The presence of anxiety or agitation alone, if associated with subjective breathlessness and severe respiratory failure, and not accompanied by alterations of consciousness or thinking, was not considered as delirium, but instead a typical manifestation of severe COVID-19. Following the everyday practice at our institution, clinical records included at least one description of patient consciousness, attention and appropriateness of thinking per day, allowing to determine whether CAM criteria were fulfilled or not.

The outcome of hospital stay (discharge, ICU admission or death) was also recorded.

### Statistical analysis

Continuous variables were expressed as median and interquartile range (IQR), after checking the non-normal distribution of values with the Kolmogorov–Smirnov test. Categorical variables were expressed as percentages. The baseline parameters were compared between patients who developed delirium during hospital stay and those who did not using Mann–Whitney test and chi-square test for crude comparisons, and analysis of covariance (ANCOVA) or binary logistic regression for age- and sex-adjusted comparisons.

Stepwise binary logistic regression with forward selection was then applied, to test which baseline clinical factors were independently associated with the development of delirium during hospital stay (dependent variable). Covariates were selected among those factors showing a significant difference between patients with and without delirium at the univariate analysis.

A multivariate logistic regression model, accounting for relevant anamnestic and clinical variables at admission, was also applied to verify whether delirium during hospital stay was independently associated with increased mortality.

The analyses were performed with the SPSS program, considering *p* values < 0.05 as statistically significant.

## Results

The study population included 852 patients (401 F, 451 M, mean age 73 ± 14), hospitalized for suspect COVID-19 in March and April, 2020. Ninety-four patients (11% of the sample, 42 F and 52 M) developed delirium during hospital stay. A comparison of the main baseline features between patients who developed delirium and patients who did not is depicted in Table 1. Namely, patients with delirium were older (median age 82, IQR 78–89, vs 75, IQR 63–84, *p* < 0.001), had less frequently cough (27% vs 47%, age- and sex-adjusted *p* < 0.01), more frequently atypical symptoms such as syncope, postural instability and thoracic pain (26% vs 16%, age- and sex-adjusted *p* = 0.02), and had lower oxygen saturation values in room air (median 90%, IQR 83–94, vs 93%, IQR 88–95, age-and sex-adjusted *p* = 0.049).

**Table 1:** Demographic, anamnestic and clinical characteristics of patients with suspect COVID-19 at hospital admission, categorized according to the development of delirium during hospital stay

	Patients without delirium (*n* 758)	Patients with delirium (*n* 94)	*p*	p*	Odds ratio (95% confidence interval)
Demography					
Age, years	75 (63–84)	82 (78–89)	** < 0.001**	–	
Female gender, *n* (%)	360 (47)	42 (45)	0.606	–	
Symptoms before emergency department arrival					
Cough, *n* (%)	350 (47)	25 (27)	** < 0.001**	**0.009**	**0.520 (0.318–0.852)**
Dyspnea, *n* (%)	404 (54)	63 (67)	**0.015**	0.165	1.391 (0.873–2.217)
Fever, *n* (%)	616 (82)	79 (84)	0.630	0.089	1.683 (0.924–3.068)
Fatigue, *n* (%)	77 (10)	3 (3)	**0.027**	0.098	0.367 (0.112–1.205)
Atypical symptoms^a^, *n* (%)	117 (16)	24 (26)	**0.015**	**0.020**	**1.859 (1.104–3.132)**
Duration of symptoms, days	7 (4–10)	6 (3–7)	0.050	0.722	
Vital signs at emergency department triage					
Temperature, degrees	36.7 (36.0–37.5)	36.4 (36.0–37.2)	**0.028**	0.257	
Peripheral oxygen saturation in room air, %	93 (88–95)	90 (83–94)	** < 0.001**	**0.049**	
Comorbidities and functional performance					
Chronic comorbidities, number	3 (1–4)	3 (2–5)	** < 0.001**	0.812	
Hypertension, *n* (%)	433 (57)	65 (69)	**0.027**	0.985	1.005 (0.619–1.632)
Diabetes, *n* (%)	156 (21)	23 (25)	0.357	0.767	0.925 (0.551–1.552)
Heart disease, *n* (%)	204 (27)	32 (34)	0.147	0.448	0.830 (0.514–1.342)
Cancer, *n* (%)	119 (16)	11 (12)	0.307	0.221	0.656 (0.334–1.289)
Chronic kidney disease, *n* (%)	59 (8)	16 (17)	**0.003**	0.099	1.685 (0.907–3.128)
COPD, *n* (%)	82 (11)	20 (21)	**0.003**	0.127	1.553 (0.882–2.736)
Stroke, *n* (%)	43 (6)	11 (12)	**0.024**	0.361	1.400 (0.680–2.882)
Epilepsy, *n* (%)	19 (3)	6 (6)	**0.036**	**0.039**	**2.845 (1.052–7.694)**
Vascular encephalopathy, *n* (%)	54 (7)	13 (14)	**0.023**	0.594	1.201 (0.612–2.358)
Dementia, *n* (%)	118 (16)	38 (40)	** < 0.001**	** < 0.001**	**2.427 (1.433–4.110)**
Autonomy in daily activities, *n* (%)	474 (63)	29 (31)	** < 0.001**	**0.020**	**0.517 (0.296–0.902)**
Chronic medications^a^					
Drugs, number	4 (1–6)	5 (3–8)	** < 0.001**	0.376	
ARB, *n* (%)	67 (9)	17 (18)	**0.008**	0.096	1.720 (0.909–3.255)
Vasodilators, *n* (%)	13 (2)	6 (7)	**0.003**	**0.009**	**4.061 (1.418–11.620)**
Anti-platelet agents, *n* (%)	222 (30)	37 (42)	**0.024**	0.704	1.095 (0.685–1.751)
Antidepressants, *n* (%)	130 (17)	27 (30)	**0.003**	0.126	1.499 (0.893–2.516)
Antipsycotics, *n* (%)	52 (7)	15 (17)	**0.001**	**0.039**	**1.982 (1.034–3.802)**
Chest CT findings^c^					
Ground-glass opacities, *n* (%)	659 (92)	85 (99)	**0.025**	**0.032**	**8.936 (1.204–66.310)**
Consolidations, *n* (%)	468 (66)	58 (67)	0.752	0.751	1.083 (0.664–1.765)
Lung parenchyma extension, %	30 (20–50)	38 (25–60)	**0.020**	0.128	
SARS-CoV-2 testing on admission^d^					
RT-PCR positive for SARS-CoV-2, *n* (%)	480 (67)	65 (75)	0.137	0.264	1.319 (0.812–2.144)

Data are shown as median and interquartile range or percentages. Crude comparisons were made with Mann–Whitney test or chi-square test, as appropriate

Significant *p* values and corresponding odds ratios are indicated in bold

*COPD* Chronic Obstructive Pulmonary Disease, *ARB *Alpha Receptor Blockers, *CT *Computed Tomography, *SARS-CoV-2 *Severe Acute Respiratory Syndrome CoronaVirus 2, *RT-PCR *Reverse Transcriptase Polymerase Chain Reaction

*p adjusted for age and sex with ANCOVA or binary logistic regression

^a^Atypical symptoms include thoracic pain, syncope and postural instability

^b^Information available for 745 patients without delirium and 89 patients with delirium

^c^Information available for 715 patients without delirium and 86 patients with delirium

^d^Information available for 739 patients without delirium and 92 patients with delirium

Patients who developed delirium had also a higher prevalence of dementia and epilepsy, and had lower functional autonomy in daily activities (31% vs 63%, age- and sex-adjusted *p* = 0.02) (Table 1).

The main laboratory tests performed on the day of admission were not significantly different between patients with and without delirium during hospital stay, except for lactate dehydrogenase (median 448 IU/L, IQR 284–572, vs 333 IU/L, IQR 250–453, age- and sex-adjusted *p* = 0.033) and PaO_2_/FiO_2_ ratio at arterial blood gas analysis (median 175, IQR 90–296, vs 257, IQR 148–357, age- and sex-adjusted *p* = 0.02) (Table 2).

**Table 2:** Laboratory characteristics of patients with suspect COVID-19 at hospital admission, categorized according to the development of delirium during hospital stay

	Patients without delirium (*n* 758)	Patients with delirium (*n* 94)	*p*	p*
Arterial blood gas analysis				
pH	7.45 (7.41–7.47)	7.43 (7.40–7.48)	0.250	0.783
HCO_3_^−^, mmol/L	25 (23–27)	24 (22–27)	0.081	0.451
pCO_2_, mmHg	36 (33–40)	36 (31–40)	0.209	0.604
pO_2_, mmHg	75 (62–94)	70 (55–88)	**0.032**	0.053
pO_2_/FiO_2_	257 (148–357)	175 (90–296)	** < 0.001**	**0.020**
Clinical chemistry and haematology				
Haemoglobin, g/dl	13.4 (12.0–14.6)	13.7 (11.9–14.8)	0.540	0.200
White blood cells, 1000/mm^3^	6.73 (5.00–9.33)	7.41 (5.43–9.97)	0.116	0.953
Platelets, 1000/mm^3^	208 (163–268)	220 (173–273)	0.198	0.132
Creatinine, mg/dl	0.9 (0.7–1.2)	1.0 (0.8–1.5)	**0.003**	0.796
Urea, mg/dl	43 (31–69)	68 (45–117)	** < 0.001**	0.051
Sodium, mEq/L	138 (135–140)	139 (135–143)	**0.020**	0.157
Potassium, mEq/L	4.0 (3.7–4.3)	4.0 (3.6–4.4)	0.842	0.233
Creatine-phosphokinase, IU/L	125 (65–236)	214 (100–514)	** < 0.001**	0.062
Lactate-dehydrogenase, IU/L	333 (250–453)	448 (284–572)	** < 0.001**	**0.033**
Aspartate aminotransferase, IU/L	43 (29–64)	53 (35–77)	**0.020**	0.157
Alanine aminotransferase, IU/L	28 (19–47)	25 (16–43)	0.273	0.901
D-Dimer, ng/ml	1020 (636–1998)	1455 (1015–4284)	** < 0.001**	0.072
INR ratio	1.21 (1.13–1.35)	1.21 (1.11–1.35)	0.895	0.389
aPTT ratio	0.98 (0.90–1.08)	0.96 (0.87–1.07)	0.162	0.490
Fibrinogen, mg/dl	596 (480–730)	581 (486–916)	0.725	0.482
C-reactive protein, mg/L	96 (41–154)	125 (63–191)	**0.005**	0.141
Procalcitonin, ng/ml	0.16 (0.07–0.48)	0.24 (0.15–0.74)	**0.002**	0.328

Data are shown as median and interquartile range. Crude comparisons were made with Mann–Whitney test, as appropriate

Significant *p* values are indicated in bold

*p adjusted for age and sex with ANCOVA

A stepwise logistic regression model with forward selection, accounting for all clinical and laboratory variables showing differences at admission between patients with or without delirium, showed that the development of delirium during hospital stay was positively associated with age, history of epilepsy, use of vasodilators and antipsychotics as chronic medications, blood urea and LDH (Table 3). The use of antipsychotic drugs was significantly associated with a history of dementia (β ± SE 3.21 ± 0.31, *p* < 0.001), and delirium superimposed on dementia was the most frequent type detected in the studied population (38 cases out of 94, 40%).

**Table 3:** Results of a stepwise logistic regression model with forward selection, testing the anamnestic and clinical factors at hospital admission independently associated with the development of delirium in a group of 852 patients with suspect COVID-19 pneumonia

	Odds ratio	95% Confidence interval	p*
Age, years	1.093	1.046–1.143	** < 0.001**
History of epilepsy	12.470	2.324–66.922	**0.003**
Chronic use of antipsychotic drugs	4.529	1.204–17.027	**0.025**
Chronic use of vasodilators	10.039	1.298–77.647	**0.027**
Urea, mg/dl	1.011	1.004–1.019	**0.003**
Lactate dehydrogenase, IU/L	1.003	1.001–1.005	**0.001**

*Other variables considered in the forward selection: gender, symptoms before Emergency Department arrival, vital signs at Emergency department triage, number of chronic comorbidities, dementia, stroke, vascular encephalopathy, hypertension, number of drugs, use of antidepressants, use of antiepileptic drugs, lung parenchyma extension of abnormalities at chest CT, blood levels of creatinine, aspartate aminotransferase, creatinephosphokinase, C-reactive protein, procalcitonin at admission

Significant *p* values are indicated in bold

Mortality was significantly higher in patients who developed delirium during hospital stay than in those who did not (57% vs 30%, age- and sex-adjusted *p* < 0.001). However, a multivariate logistic regression model, accounting for multiple possible confounders, showed that delirium was not independently associated with mortality (Table 4).

**Table 4:** Results of a multivariate logistic regression model, exploring the factors associated with mortality in the studied population of 852 patients admitted with suspect COVID-19 pneumonia

	Odds ratio	95% Confidence interval	*p*
Age, years	1.070	1.035–1.105	** < 0.001**
Number of chronic comorbidities	1.193	1.014–1.403	**0.034**
PaO_2_/FiO_2_ at admission	0.990	0.986–0.994	** < 0.001**
Lactate dehydrogenase at admission, IU/L	1.003	1.001–1.006	**0.005**
Gender (female vs male)	0.851	0.454–1.598	0.616
Symptom duration before visit, days	0.945	0.883–1.012	0.106
Chest CT visual scoring extension, %	1.003	0.985–1.020	0.776
D-dimer at admission, ng/ml	1.000	1.000–1.000	0.429
C-reactive protein at admission, mg/L	1.002	0.997–1.007	0.443
Creatinine at admission, mg/dl	1.362	0.890–2.085	0.154
Delirium during hospital stay	1.231	0.554–2.738	0.610

Significant *p* values are indicated in bold

## Discussion

Delirium represents a common complication of hospital stay in patients with moderate and severe COVID-19. In our population, delirium was associated with older age, neurologic comorbidities including dementia and epilepsy, atypical symptoms of COVID-19 and worse gas exchange at the moment of admission. Delirium was also associated with dramatic increase in mortality, but this association was not independent of respiratory conditions.

This study represents one of the earliest reports on the epidemiology and clinical correlations of delirium in moderate and severe COVID-19. The findings confirm the assumption that delirium is a common complication of hospital stay during the clinical course of COVID-19, especially in older patients with neurologic comorbidities [13]. Thus, diagnosis and adequate management of delirium should be a fundamental part of clinical protocols for the care of patients with moderate or severe forms of COVID-19.

The incidence of delirium in our population was, however, lower (11%) than that predicted for COVID-19 [13] and that previously reported in large multicenter studies conducted in the pre-COVID era in both general medical wards and ICUs (> 20%) [4, 20]. There are, however, important fluctuations in the reported incidence of delirium among different clinical settings and populations of hospitalized patients, and an incidence of 11% is similar to that previously reported in a multicenter study of internal medicine wards in Italy [21]. On the other side, studies conducted in an ICU setting showed that patients with critical illness and respiratory failure due to sepsis have an incidence of delirium up to 70% [22]. This difference can probably be explained by the different demography of patients hospitalized during the COVID-19 pandemic, that were in average younger, less disabled and with lower number of chronic comorbidities than patients that are generally admitted to medical and geriatric wards [4]. Some atypical characteristics of the respiratory failure associated with COVID-19 pneumonia, with relatively low prevalence of subjective dyspnea in spite of impaired respiratory exchanges, could also be involved [23].

The context of the COVID-19 pandemic peak, with a large number of patients seeking hospital care in a limited time lapse, should also be considered as a factor favoring underreporting of delirium, especially when the admission is not performed in geriatric wards. In such contexts, a comprehensive geriatric assessment is hardly ever performed, and may be unfeasible in case of extreme patient overflow. This is also the main reason why prevalent delirium, i.e., already present on patient admission, was not a focus of the present study, since data on patients’ mental status at ED evaluation before ward admission were unavailable for the majority of participants.

However, patients with severe forms of COVID-19 pneumonia may also experience agitation as a direct consequence of breathlessness and fever [24]. These manifestations, if not associated with altered consciousness and disorganized thinking, do not represent delirium and do not fulfil CAM criteria, but require the administration of sedative drugs increasing themselves the risk of delirium [22]. Therefore, an accurate assessment of delirium in severe COVID-19 patients can be challenging, and this circumstances should be considered as the main limitation of the present retrospective study.

Moreover, the CAM criteria have been validated as a live assessment tool and not for retrospectively evaluating clinical records. The CAM criteria have already been used for a retrospective evaluation of the presence of delirium in nursing homes, but in this setting many more neuropsychological variables were available [25]. Thus, the peculiar methodology of delirium assessment could also have contributed to its underreporting in the present investigation.

The association of delirium with older age and neurologic comorbidities, namely dementia, is well known in the literature, and the diagnostic category of delirium superimposed on dementia is being increasingly studied [26, 27]. COVID-19-associated respiratory failure and, possibly, direct infection of the central nervous system by SARS-CoV-2, may be particularly effective in unmasking the deliriogenic potential of dementia [8]. This situation may pose further diagnostic dilemmas, since the clinical manifestations of delirium superimposed on dementia are sometimes difficult to distinguish from the usual symptoms of dementia, especially in clinical contexts, where the personnel lacks geriatric training [26, 27]. Moreover, the treatment of delirium superimposed on dementia in COVID-19 patients should be evaluated carefully, since many sedative drugs may contribute to worsen the respiratory failure and interact with QT-prolonging drugs, such as hydroxychloroquine and azithromycin, that are often prescribed for the treatment of SARS-COV-2 infection [28].

The association of serum lactate-dehydrogenase and urea with delirium in COVID-19 underlines the importance of peripheral perfusion and dehydration as precipitating factors of delirium. In severe forms of COVID-19 pneumonia, a viral sepsis may occur, with impaired perfusion of organs including the central nervous system [29]. This pathophysiological mechanism may be involved also in the development of delirium, and should be carefully considered when taking care for COVID-19 patients. Additionally, the COVID-19 pneumonia is associated with a high risk of dehydration and pre-renal acute kidney failure, due to negative fluid balance caused by fever, tachypnea and oxygen supply. The association of dehydration with delirium is well established in elderly patients [30], and fluid balance should be optimized in COVID-19 pneumonia also to avoid the development of delirium.

In ordinary practice, delirium is associated with increased risk for adverse outcomes [16]. Despite in our case series incident delirium was indeed associated with a significantly higher mortality, this association was not independent of age, multimorbidity and baseline respiratory conditions (Table 4). From this perspective, in patients with COVID-19 pneumonia, delirium should be considered as a marker of disease severity, rather than an independent predictor of death. However, the possible additive effect of incident delirium on mortality could also have been masked by the strong association of age and severe COVID-19 clinical presentation with both incident delirium and mortality. Thus, in older patients with severe COVID-19, preventive measures to avoid the onset of delirium should be made effective. Older patients with COVID-19 pneumonia should ideally be treated in COVID units with geriatric expertise [31, 32]. Geriatric expertise is in fact extremely important for an adequate management of complications that are typical of older patients even in the context of a novel infectious disease such as COVID-19.

Besides the issues that could have led to delirium underreporting, some other limitations of our study should be additionally considered when interpreting results. The retrospective design of the study and the emergency context of patient care prevented the collection of complete data regarding delirium subtypes, timing of onset and therapeutic strategies. It was also not possible to test associations between sedative drugs administered during hospital stay and the development of delirium. Patients with cognitive impairment and those who experienced delirium may also have reported previous symptoms related to COVID-19 in incomplete way. Finally, a formal comprehensive geriatric assessment, considering the cognitive conditions of patients, was not performed due to the elevated flows of patients that were admitted during the pandemic peak.

In spite of these limitations, this study represents one of the first reports of delirium epidemiology and clinical correlates in a large group of patients hospitalized with COVID-19 pneumonia, confirming that delirium is one of the main complications of the severe forms of this novel disease. Future studies, with sounder design, should better evaluate the clinical characteristics of patients developing delirium during COVID-19 pneumonia and the possible management strategies.

## Conclusions

In patients hospitalized with suspect COVID-19 pneumonia during the pandemic peak, delirium was a common complication of stay, associated with older age, neurological comorbidities and higher serum urea and lactate-dehydrogenase levels at admission. Delirium was associated with a higher mortality, but this association was not independent of age and respiratory exchanges at admission, making it a marker of severe COVID-19, rather than a prognostic factor. Clinicians caring for COVID-19 patients should be aware of the risk of delirium and be trained into its optimal management.
